# Electrophysiological and morphological modulation of neuronal-glial network by breast cancer and nontumorigenic mammary cell conditioned medium

**DOI:** 10.3389/fbioe.2024.1368851

**Published:** 2024-04-04

**Authors:** Donatella Di Lisa, Katia Cortese, Michela Chiappalone, Pietro Arnaldi, Sergio Martinoia, Patrizio Castagnola, Laura Pastorino

**Affiliations:** ^1^ Department of Informatics, Bioengineering, Robotics and Systems Engineering, University of Genoa, Genoa, Italy; ^2^ Department of Experimental Medicine, Cellular Electron Microscopy Lab, University of Genoa, Genova, Italy; ^3^ IRCCS Ospedale Policlinico San Martino, Genova, Italy; ^4^ RAISE Ecosystem, Genova, Italy

**Keywords:** breast cancer, HER2/ERBB2, secretome, tumor progression, human induced pluripotent stem cells, electrophysiological activity

## Abstract

Breast cancer is a significant global health concern, with the overexpression of human epidermal growth factor receptor 2 (HER2/ERBB2) being a driver oncogene in 20%–30% of cases. Indeed, HER2/ERBB2 plays a crucial role in regulating cell growth, differentiation, and survival via a complex signaling network. Overexpression of HER2/ERBB2 is associated with more aggressive behavior and increased risk of brain metastases, which remains a significant clinical challenge for treatment. Recent research has highlighted the role of breast cancer secretomes in promoting tumor progression, including excessive proliferation, immune invasion, and resistance to anti-cancer therapy, and their potential as cancer biomarkers. In this study, we investigated the impact of ERBB2+ breast cancer *SKBR-3* cell line compared with *MCF10-A* mammary non-tumorigenic cell *conditioned medium* on the electrophysiological activity and morphology of neural networks derived from neurons differentiated from human induced pluripotent stem cells. Our findings provide evidence of active modulation of neuronal-glial networks by *SKBR-*3 and *MCF10-A conditioned medium.* These results provide insights into the complex interactions between breast cancer cells and the surrounding microenvironment. Further research is necessary to identify the specific factors within breast cancer *conditioned medium* that mediate these effects and to develop targeted therapies that disrupt this interaction.

## 1 Introduction

Human epidermal growth factor receptor 2 (ERBB2/HER2) is a member of the human epidermal growth factor receptor (HER) family. It plays a key role in the ERBB/HER family, cooperating with other ERBB/HER receptors via a complex signaling network to regulate cell growth, differentiation, and survival. The ERBB2/HER2 proto-oncogene (also known as neu or c-erbB-2) encodes the transmembrane ERBB2/HER2 protein. ERBB2/HER2 is normally expressed in several cell and tissue types excluding those of hematopoietic origin (Press et al., 1990), and, importantly, is frequently overexpressed in a number of human cancers (Slamon et al., 1987).

The HER receptor family is involved in the regulation of normal breast growth and development (Zahnow, 2006), and overexpression of ERBB2/HER2 is associated with breast cancer (Krishnamurti and Silverman, 2014). Breast cancer is a significant health concern worldwide, with the amplification of the ERBB2/HER2/Neu gene being a feature found in 20%–30% of cases.

Breast cancer was classified until 2018 into four main molecular subtypes: Luminal A, Luminal B, Basal like/triple negative, and HER2-enriched. ERBB2/HER2-low breast cancer is a novel category introduced in 2018. (Lin et al., 2019).

ERBB2/HER2-low breast cancer is considered a subtype of hormone receptor-positive, HER2 positive but not amplified breast cancer and is being treated in a similar way to the HER2-enriched subtype (Marchiò et al., 2021). Distinct molecular subtypes of breast cancers have been associated with specific gene expression signatures that are linked to different risks for brain metastases development. (Horr and Buechler, 2021; Cosgrove et al., 2022). This suggests that cancer cells possess unique molecular programs that give them intrinsic advantages for survival in the foreign tissue of the brain. Overexpression of HER2/ERBB2 is associated with increased risk of brain metastases (Stavrou et al., 2021). Indeed, asymptomatic brain metastases have been found in up to 12% of early-stage ERBB2+ breast cancer, while 25%–50% of patients with metastatic breast cancer (MBC) will develop brain metastases (BM) (Zimmer et al., 2022). Breast cancer brain metastases (BCBMs) are an increasingly common occurrence and portend a poor prognosis. Understanding how breast cancer cells reach specifically the brain, whether and how they prepare the brain for their arrival, what factors optimize BCBM cells to seed and survive in the brain microenvironment will be crucial for the development of efficient treatments for brain metastatic and resistant tumors. In fact, treatment of BCBMs represents an unmet medical need as there are currently no approved therapies aside from surgical resection and cranial radiation.

Recent research has shown that the biophysical and mechanical properties of the brain extracellular matrix (ECM) and brain resident cells play a critical role in creating optimal conditions for the survival, colonization, and outgrowth of breast cancer cells in this distinct microenvironment (Carvalho et al., 2020). In the last years, it has become increasingly evident that cancer cells release several types of biomolecules in the extracellular environment. In particular, secreted proteins or protein shedded from the plasma membrane or associated to extracellular vesicles (EVs), collectively termed as cancer secretome, are promising cancer biomarkers since they might be detectable in blood or other biofluids (Pavlou and Diamandis, 2010; Veyssière et al., 2022). Cancer secretomes seem to play an important role in enhancing proliferation, reducing apoptosis, immune invasion, alteration in energy metabolism, and development of resistance against anti-cancer therapy (Patel et al., 2014). Furthermore, is has been shown that the cancer secretome in part represents the tumor microenvironment that plays a key role in tumor promoting processes such as angiogenesis and invasion (Jiang et al., 2020).

Recently, several studies have been focused to uncover and characterize the secretome of cancer cells aiming to identify biomarkers of metastatization. Indeed, there is a plethora of evidence demonstrating that cancer cells prepare the metastatic organ in the early stages of disease progression; long before intravasation, communicating with distant tissues or organs, mainly by secreting soluble factors and/or extracellular vesicles (EV) that activate resident cells, immune cells and promote ECM remodeling, priming a permissive pre-metastatic niche. Among the breast cancer subtypes, HER2-enriched (HER2+) and triple-negative primary tumors show higher rates of brain relapse relative to hormone receptor-positive tumors, suggesting that some innate feature(s) of primary tumor cells may dictate the development of brain metastases. ERBB2+ breast cancer cells secrete EVs, and their number and content are largely modulated by ERBB2+ targeted therapy (Marconi et al., 2021; Santamaria et al., 2021). Recent studies in the field indicate that prior to reaching the brain, certain metastatic primary breast cancer cells are already equipped with proteins necessary for the formation of brain metastases. These proteins include serpins, matrix metalloproteases, and αB-crystallin. Once these cells enter the brain, recent research has shown that they can transform nearby glial cells and neurons, creating a more conducive environment for their growth. It is worth noting that tumor-activated astrocytes have been identified as having a protective role against brain metastases and enhance the proliferation and survival of cancer cells. Targeting these reactive astrocytes within the tumor microenvironment has demonstrated therapeutic benefits *in vivo*, reducing the growth of cancer cells in the brain. Improvements in the therapy of ERBB2+ breast cancer have led to extended survival in patients with advanced disease, but the prevention and treatment of central nervous system metastases still pose unique clinical challenges (Zimmer et al., 2022). Breast cancer is one of the most likely causes of brain tumor-derived epilepsy and this condition severely impacts patients’ quality of life (Asano et al., 2022). However, little is known about whether and how ERBB2+ cancer cell secretomes activate and/or modify the spontaneous electrophysiological activity and the morphology of the neuronal network to support cancer progression.

In this work, we aimed at investigating whether the conditioned medium collected from *SKBR-3* cultures, a widely used ERBB2+ human breast cancer cell line, and from *MCF10-A*, a non-tumorigenic human mammary cell line, impact on the modulation of the electrophysiological activity and on the morphology of neural networks. To this end, we used cultures of neurons differentiated from human induced pluripotent stem cells (h-iPSCs) co-cultured with rat astrocytes, grown onto Micro-Electrode Arrays (MEAs) to allow their electrophysiological monitoring over time (Chiappalone et al., 2006). The obtained results showed that both breast-derived conditioned medium from ERBB2+ *SKBR-3* cells and from non-tumorigenic *MCF10-A* strongly affected neural activity as well as neurons and astrocytes morphology in distinct ways, providing a proof-of principle evidence in support of a key role for the conditioned medium in the active modulation of neuronal networks.

## 2 Materials and methods

### 2.1 Human induced pluripotent stem cells and neuronal differentiation protocol

h-iPSCs were generated by introducing lentiviral transduction to fibroblasts derived from a healthy donor. The cells used in this study were generously provided by Frega et al. The complete protocol for generating and maintaining the rtTa/NgN2 positive cell line has been previously published (Chiappalone et al., 2019). Once the h-iPSCs reached 80% confluence, they were detached and seeded as individual cells in 6-well plates that were pre-coated with Matrigel solution. The cells were cultured in Essential 8 Flex Medium (Cat. A2858501, Gibco ThemoFisher) supplemented with 1% penicillin/streptomycin (Cat. 15140122, Gibco. ThermoFisher), 50 μg/mLG418 (Cat. G8168, Merck Life Science), 0.5 μg/mL puromycin (Cat. P8833, Merck Life Science), and 4 μg/mL Doxycycline. The differentiation process into neurons was induced by adding Doxycycline to the medium, and this step was designated as Day After Differentiation 0 (DAD 0). On DAD 1, the medium was replaced with DMEM/F12 (Cat. 11320074, Gibco, ThemoFisher) supplemented with 1% N2-supplement (Cat. 17502048, Gibco, ThermoFisher), 1% MEM non-essential amino acid solution (Cat. 11140050, Gibco, ThermoFisher), 1% penicillin/streptomycin, 10 μg/mL human BDNF (Cat. PHC7074, Gibco, ThermoFisher), 10 μg/mL human NT-3 (Cat. SRP312, Merck Life Science), and 4 μg/mLdoxycycline. At DAD 3, the neurons were detached using Accutase (Cat. A6964, Sigma Aldrich, Merck Life Science), collected in Neurobasal medium supplemented with 1% penicillin/streptomycin, 2% B27 supplement (Cat. 17504,044, Gibco, ThermoFisher), 1% glutamax (Cat. 35050038, Gibco, ThermoFisher), 10 μg/mL human BDNF, 10 μg/mL human NT-3, and 4 μg/mL doxycycline and centrifuged at 1,200 rpm for 5 min. The cells were then resuspended in complete Neurobasal medium and were ready for use.

### 2.2 Astrocyte culture

Astrocytes were obtained from isolated cortices from dissection of E18 rat, and non-astrocytic cells were removed thanks to 6 h of orbital shaking after plating. The specific procedures and methods used in this process were previously described in the study by Aprile et al. (2019). Astrocytes were placed in T-75 flasks containing DMEM High Glucose supplemented (Cat. 41965039, Gibco, ThermoFisher) with 10% FBS (Cat. 10270106, Gibco Invitrogen) and 1% penicillin/streptomycin. The flasks were then incubated at 37°C with 5% CO2, and the medium was replaced every 3 days for a period of 7–10 days.

### 2.3 Human breast cell culture and preparation of conditioned medium

Human ERBB2+/SKBR3 breast cancer cell lines and MCF10 mammary epithelial cells were obtained from Biological Bank of IRCCS Ospedale Policlinico San Martino, Genova.

SKBR-3 were cultured and maintained in DMEM medium supplemented with 10% FBS (fetal bovine serum), 1 mM glutamine (Cat. C0550-100, Voden medical instruments), 10 μg/mL insulin (Cat. I0516, Sigma Aldrich, Merck Life Science), 10 ug/mL penicillin, and 10 µg/streptomycin. *MCF10-A* cells were cultured I DMEM/F12 with 5% horse serum (Cat. H1270, Sigma Aldrich, Merck Life Science) 20 ng/mL EGF (Cat. 78006.1, Voden medical instruments), 0.5 mg/mL hydrocortisone (Cat. 07925, Voden medical instruments), 100 ng/mL cholera toxin (Cat. C8052, Sigma Aldrich, Merck Life Science) 10 μg/mL insulin, 10 ug/mL penicillin, and 10 μg/mL streptomycin. Cells were maintained in humidified incubator at 37°C and 5% CO_2_. When about 80% confluence was obtained, the medium has been exchanged for *conditioning medium* based on Neurobasal medium supplemented with 2% B27, glutamine 1 mM, 10 ug/mL penicillin, and 10 streptomycin. After 48 h, the *conditioning media* exposed to both cell types (referred as *conditioned medium*) were collected and filtered through a 0.2 µm filter and stored at −20°C until use. After removal of the conditioning medium, cells were subjected to a viability assay (NUCLEAR-ID^®^ Blue/Red cell viability reagent, Prod. No. ENZ-53005, Enzo Life Sciences) for 30 min of incubation, before being washed with PBS and observed qualitatively under a fluorescence microscope (Olympus IX70 wide field microscope equipped with Hamamatsu camera Orca-Flash 4.0 V3/LT+). Consequently, cells were trypsinized and detached to quantify the portion of live cells *versus* the apoptotic ones.

### 2.4 2D neuronal cell culture

2D neuronal culture was obtained by seeding and co-culturing neurons differentiated from h-IPSCs and primary rat astrocytes onto multi-electrode array (MEA) devices. The day before seeding, MEAs were assembled with donuts-shaped Poly-dimethyl-siloxane (PDMS) structures (internal and external diameters: 5 and 22 mm respectively) to confine the active electrodes area. MEAs were sterilized in oven at 120° for 2 h. The active area of MEAs were functionalized with 1% chitosan solution pre-sterilized by autoclaving it at 120°C and left in the incubator overnight at 37°C, (Di Lisa et al., 2022). The coating solution was removed from the MEA which was then washed twice with water and left to dry under the laminar hood until seeding. Cells were seeded at a cell density of 1,500 cell/mm^2^. After 2h, complete Neurobasal medium was added and cells were left in incubator at 37°C, 5% CO_2_ and 95% humidity for 57 days. Starting from DIV11, 2.5% FBS solution has been added to the complete Neurobasal medium, and from DIV14, doxycycline has been removed from the complete Neurobasal medium (referred as *iNeurons medium*). *iNeurons medium* was based on Neurobasal medium supplemented with 1% penicillin/streptomycin, 2% B27 supplement, 1% glutamax, 10 μg/mL human BDNF, 10 μg/mL human NT-3, and 2.5% FBS.

### 2.5 MEA recording and analysis

#### 2.5.1 Recording protocol

The spontaneous electrophysiological activity of the neuronal network was recorded by using Micro-Electrode Arrays–MEAs. MEAs, after pioneering works between the 80 s and the 90 s (Gross et al., 1992), have become a standard in the field of *in vitro* electrophysiology. Specifically, two primary characteristics render MEAs a valuable asset for electrophysiological assessments: firstly, their non-invasiveness enables prolonged recordings, facilitating long-term observation; and secondly, they enable multi-site recordings, enhancing data collection capabilities. The devices we used for our study are commercial (MultiChannel System, MCS, Reutlingen, Germany) and consist of 60 planar microelectrodes (TiN/SiN, 30 μm electrode diameter, 200 μm spaced) arranged over an 8 × 8 square grid (except the corners). Considering that one of the electrodes is used as the reference one, we could rely on a total of 59 recording electrodes for our acquisitions.

Electrophysiological recordings were carried out at different time points. The first recording was carried out at DIV 41 (*Starting point* -*D0*), after that, 2D neuronal networks were exposed to the *conditioning medium*. The *conditioned medium* was replaced with fresh medium, with a 50% replacement, every 2–3 days during the experiment. Recording sessions were conducted at specific time points, namely, DIV 43, 46, 48, 50, 53, 55, and 57 which were referred to *D2*, *D5*, *D7*, *D9*, *D12*, *D14*, and *D16*, respectively. Control samples were cultured performing a half-medium change every 2–3 days using *conditioning medium*. Recording sessions were carried out acquiring 20 min of spontaneous electrophysiological activity using the MEA 2100-System (MCS), with a sampling rate of 10 kHz. During the electrophysiological recordings, the cell culture was exposed to a continuous slow flow of humidified gas (air supplemented with 5.5% CO_2_) at 37°C to maintain incubator-like conditions.

#### 2.5.2 Data analysis

Extracellularly recorded signals are characterized by two typical modes of firing: spikes (i.e., suprathreshold events related to the action potentials of the neurons) and bursts (a fast sequence of spikes). The analysis pipeline to extract the parameters of interest related to both spikes and bursts is briefly reported. Initially, a Spike Detection algorithm was executed, employing the Precision Timing Spike Detection (PTSD) method (Maccione et al., 2009). Upon defining a threshold (set at 6 times the standard deviation of the noise, calculated separately for each channel), a spike was identified when the difference between the signal’s maximum and minimum values exceeded the threshold. Afterwards, the Burst Detection was performed: bursts were defined as a sequence of at least five spikes having an ISI (inter-spike interval, i.e., time intervals between two consecutive spikes) smaller than a reference value (set at 100 m) (Pasquale et al., 2010). Upon detecting spikes and bursts, we computed the following metrics:

• Mean Firing Rate–MFR (spikes/s): it was calculated as the total number of spikes detected on a channel divided by the duration of the recording.
• Mean Bursting Rate–MBR (burst/min): it is analogous of the previous parameter but computed over bursts (which are less frequent than the spikes, and this explains why we consider as unit of time the minutes instead of the seconds).
• Mean Burst Duration (MBD, ms): it indicates the average duration of the bursts at the single channel level.
• Percentage of Random Spikes (PRS, %): it indicates the number of spikes not fired within a burst divided by the total number of detected spikes in a channel.

To investigate and compare all the 2D cell culture conditions, the different parameters values were extracted, normalized with respect to the value at D0 and averaged across the same experimental conditions. The above analyses have been performed by means of the *SpyCode* software package developed in MATLAB (MathWorks, Natick, MA, USA) by our group (Bologna et al., 2010), together with custom developed scripts, as well in MATLAB.

### 2.6 Immunostaining

After the recording session, 2D neuronal cultures were fixed with 4% paraformaldehyde for 20 min at room temperature. Permeabilization was achieved with phosphate buffer solution (PBS, Cat. 18912014, Gibco, ThermoFisher) containing 0.2% Triton-X100 (Cat. X100, Merck Life Science) for 15 min at room temperature. To prevent non-specific binding of antibodies, cells were then incubated in a blocking buffer solution (BBS) consisting of PBS, 0.3% bovine serum albumin (BSA, Cat. A9418, Merck Life Science), and 0.5% fetal bovine serum (FBS) for 35 min at RT. Cultures were incubated with primary antibody diluted in BBS for 1 h at room temperature. Cultures were rinsed two times with PBS and finally exposed to secondary antibodies. MAP-2 (dendritic microtubule-associated protein, Cat. 188 002 and 188,011, Synaptic System) and GFAP (glial fibrillary acidic protein, Cat. 173 002 and 173 01, Synaptic System) were used as primary antibodies to evaluate the morphology of neuronal and glial cells, respectively; Dapi (10 μg/mL, Cat. 75004, Voden medical instruments) was used to label nuclei. Alexa Fluor 488 and Alexa Fluor 549 Goat anti mouse or Goat anti rabbit were used as a secondary antibody (Cat. A11001, A11003, A11008, A11035, Gibco, ThermoFisher). An Olympus BX-51 upright microscope was used for immunofluorescence and the image acquisition was done with a Hamamatsu Orca ER II digital cooled CCD camera driven by *Image ProPlus* software (Media Cybernetic).

### 2.7 Electron microscopy

2D neuronal culture cells (DIV 57) were washed out in 0.1 M cacodylate buffer and fixed in 0.1 M cacodylate buffer containing 2.5% glutaraldehyde (Electron Microscopy Science, Hatfield, PA, United States), for 1 h at room temperature. The cells were postfixed in 1% osmium tetroxide for 2 h and 1% aqueous uranyl acetate for 1 h. Subsequently, samples were dehydrated through a graded ethanol series and flat embedded in epoxy resin (Poly-Bed; Polysciences, Inc., Warrington, PA) for 24 h at 60C. Ultrathin sections (50 nm) were cut parallel to the substrate and counterstained with 5% uranyl acetate in 50% ethanol. Electron micrographs were acquired as single snapshots and MIA (multiple image alignment) at Hitachi 7,800 120Kv electron microscope (Hitachi, Tokyo, Japan) equipped with a Megaview III digital camera and Radius software (EMSIS, Muenster, Germany).

### 2.8 Statistical analysis

Regarding electrophysiological characterization, to evaluate statistically significant differences between control and the conditioned cultures, statistical analysis was performed using the 2way-ANOVA’s test followed by Tukey’s multiple comparisons, since data follow a normal distribution (evaluated by the Kolmogorov-Smirnov normality test): (*) *p* ≤ 0.0001, significant statistical differences between different groups at specific time points; significant statistical differences within group during different time points: *control* group (■) *p* ≤ 0.0001, *SKBR-3* group (•) *p* ≤ 0.0001 and *MCF10-A* (^▲^) *p* ≤ 0.0001. For immunofluorescence characterization, to evaluate statistically significant differences between control and the conditioned cultures, statistical analysis was performed using the 1way-ANOVA’s test followed by Tukey’s multiple comparison: (*) *p* ≤ 0.01, (**) *p* ≤ 0.001 and (***) *p* ≤ 0.001.

## 3 Results

### 3.1 Electrophysiological characterization

Before starting this study, the effects of the *conditioning medium* on the *MCF10-A* and *SKBR-3* cell lines were evaluated by a viability assay performed after 48 h of exposure to *iNeurons medium*. The results showed that the portion of dead cells was marginal (<4%) for both cell lines, without significant observable differences compared to the standard culture medium, demonstrating the adequacy of the conditioning protocol (Supplementary Figure S1).

**FIGURE 1:**
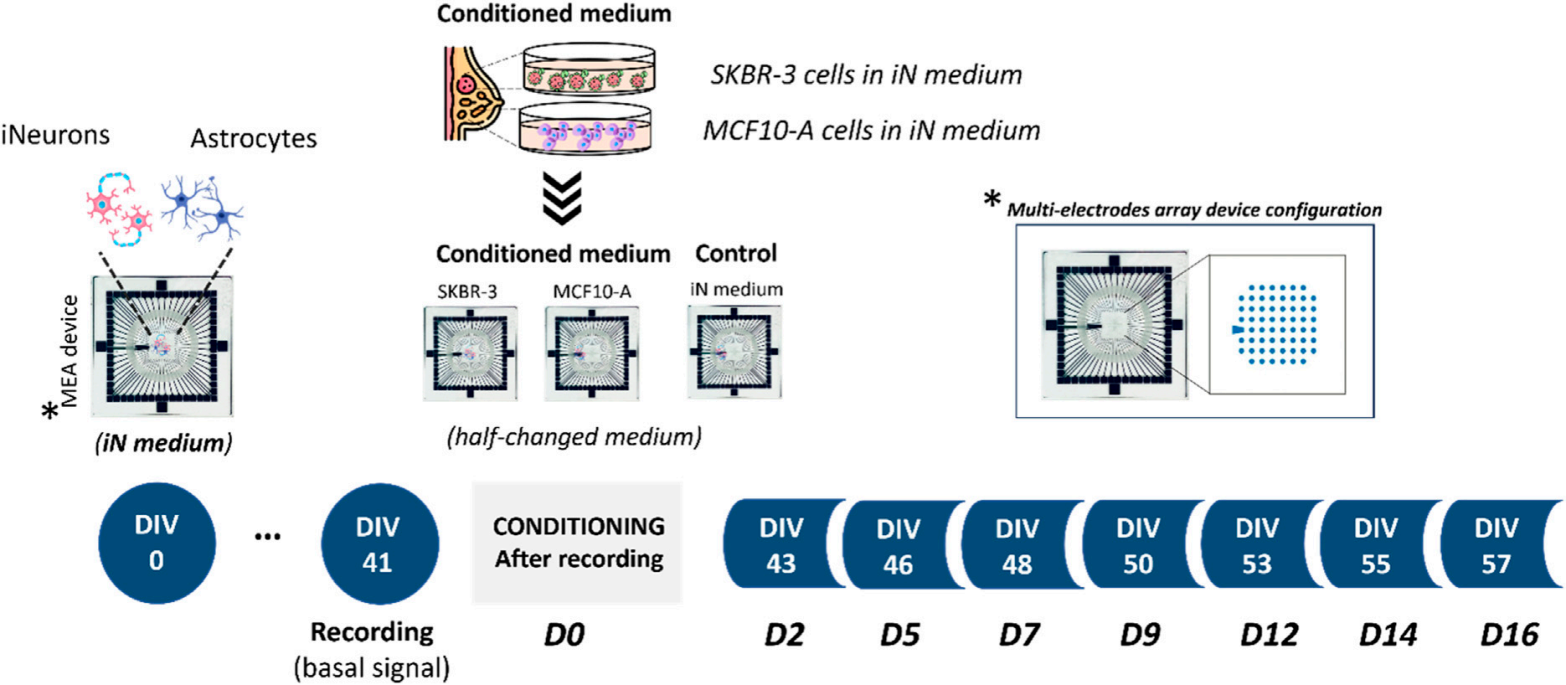
Experimental design. Schematics showing co-culture of human induced pluripotent stem cell (*hiPSC*)-derived neurons with primary astrocytes onto Micro-Electrode Arrays (MEAs). Experimental protocol involved the conditioning process starting from DIV41; specifically, three different culture conditions were investigated: 2D neural networks exposed to iN medium and 2D neural networks exposed to culture medium previously exposed to *SKBR-3* and *MCF10-A* cells. MEA is an arrangement of typically 60 electrodes aligned in an 8 × 8 grid, allowing the targeting of several sites in parallel for extracellular recording.

The impact of *SKBR-3* and *MCF10-A* cell *conditioned medium* on the activity of nervous cells was then characterized by recording the spontaneous activity of mature neuronal networks before and after their exposure to *conditioned medium*. To this purpose, neurons differentiated from human induced pluripotent stem cells were co-cultured with astrocytes onto MEA devices until DIV 41, when mature functional neuronal networks were formed, then the networks were exposed either to *SKBR-3* or *MCF10-A conditioned medium* for up to 2 weeks. At the same time, neuronal networks cultured using *iNeurons medium* were considered as controls (Figure 1).

**FIGURE 2:**
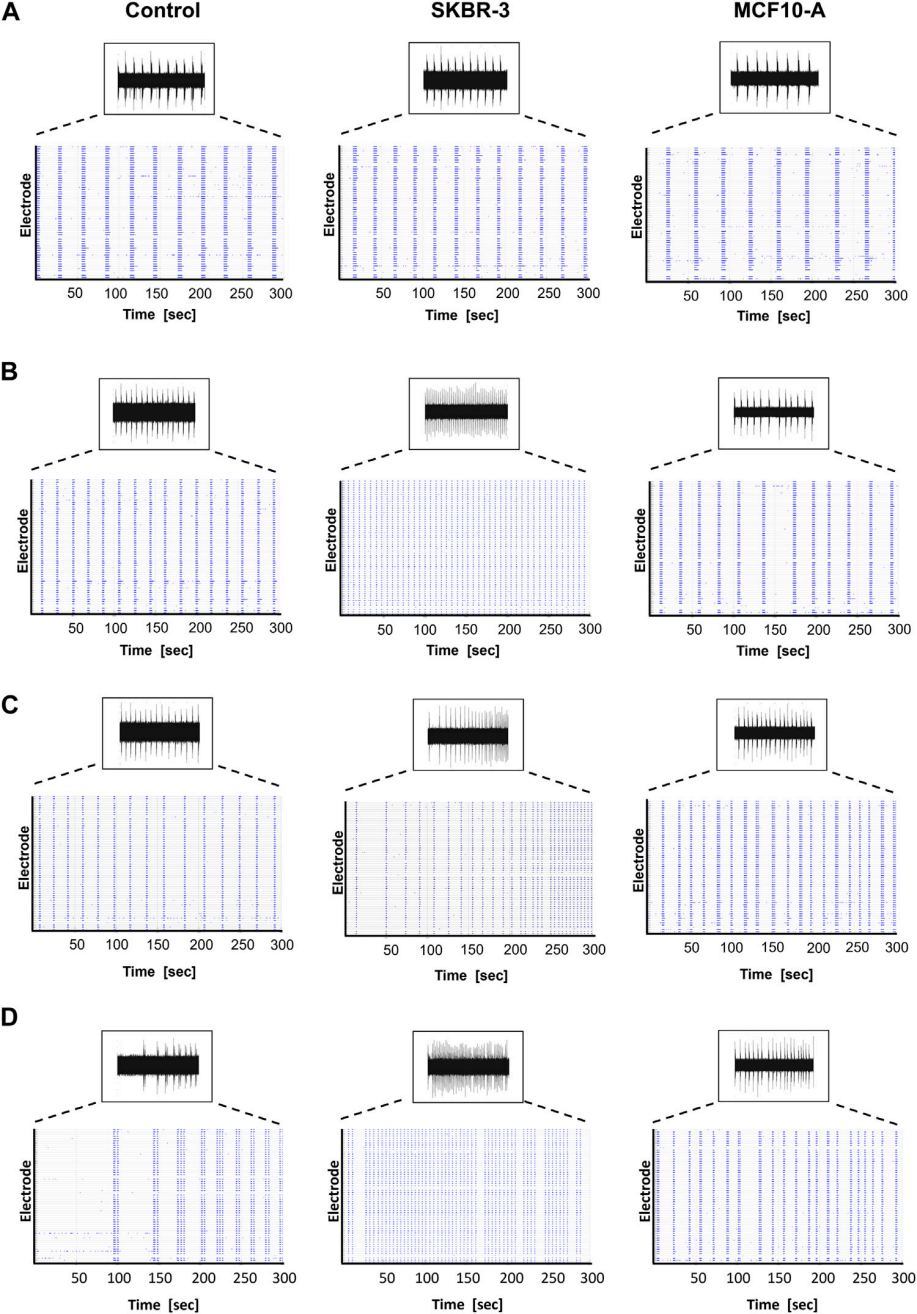
Spontaneous electrophysiological activity of control, *SKBR-3* and *MCF10-A* conditioned neuronal networks. Representative *raw data* from one channel and *raster plot* (i.e., spikes) from all the recording channels during a 300 s-long recording, exhibiting the spontaneous electrophysiological activity at different time points during development **(A)** *D0*, **(B)** *D5*, **(C)** *D9*, **(D)** *D16*. A raster plot is a qualitative representation of recording activity in which each blue dot represents a detected spike at the given electrode index (*y*-axis) at specific time points (*x*-axis).

After recording the basal spontaneous activity at DIV41, the conditioning protocol was started, and the spontaneous activity of the neuronal networks was recorded at different timepoints to observe the effect of *conditioned medium* on the neural functional behavior over time. Namely, *SKBR-3* conditioned networks (n° samples = 4), *MCF10-A* conditioned networks (n° samples = 4) and 2D control networks (n° samples = 4) were characterized in terms of spontaneous electrophysiological activity. Figure 2 shows the spontaneous activity (raw signal) and the global electrophysiological behavior of representative networks for each condition and each timepoint. Specifically, electrophysiological activity is qualitatively shown in the raster plot, where 300s of spontaneous activity are displayed. In all experimental conditions, at *D0* (the first day of recording), samples showed synchronous bursts, that displayed comparable patterns typically observed in mature neuronal networks (Figure 2A). By *D5*, which corresponds to 5 days of conditioning protocol, networks conditioned with *SKBR-3 conditioned medium* began to display functional modifications, as shown in Figure 2B. The network activity significantly increased and was characterized by a high frequency of synchronous bursts with short duration. Meanwhile, the *control* networks and the samples conditioned with *MCF10-A conditioned medium* showed patterns similar to those observed at *D0*. Moving forward to *D9*, the *SKBR-3* conditioned networks exhibited further evolution in activity (Figure 2C) The *MCF10*-A conditioned networks also exhibited a variation in firing frequency, showing a similar increase compared to the *control* group. Finally, raster plots generated on the final day of conditioning (*D16*) confirmed the functional changes observed in the *SKBR-3* networks (Figure 2D). On the other hand, the *MCF10-A* conditioned networks and *control* exhibited patterns consistent with relatively minimal evolution.

Anomalies in activity patterns were also observed for *SKBR-3* conditioned networks. Namely, some samples were characterized by a very rapid and synchronous network phenomena, others showed disorganized and uncoordinated spiking activity (Supplementary Figure S2).

Subsequently, a quantitative analysis of the data was performed, focusing on the evaluation of main electrophysiological parameters such as mean firing rate (MFR), mean burst rate (MBR), mean burst duration (MBD) and percentage of random spikes (%RS). These parameters were evaluated, normalized with respect to the value at D0 and averaged across the same experimental conditions, to assess the network dynamics and provide quantitative insights into the observed phenomena.

**FIGURE 3:**
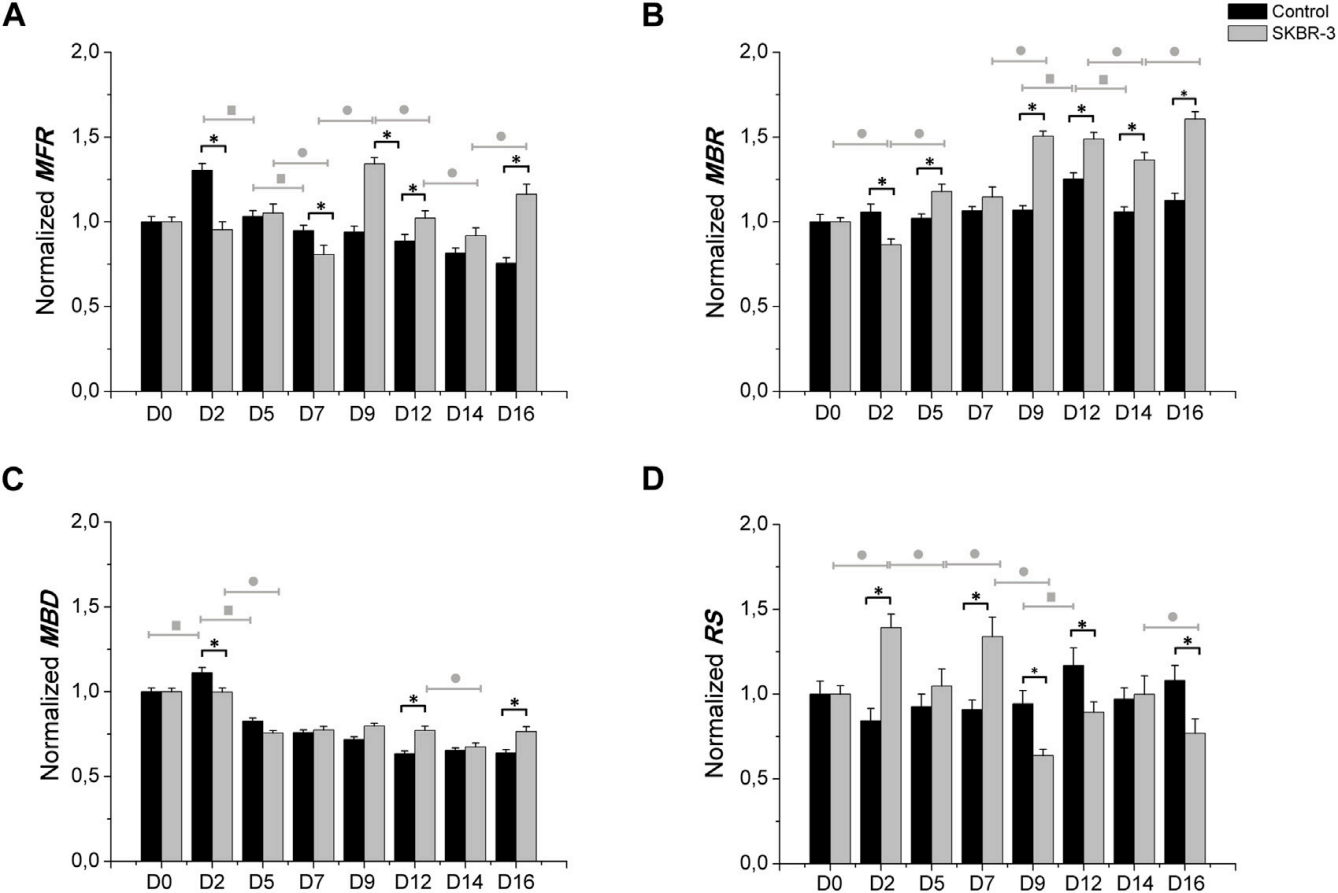
Electrophysiological characterization. MFR **(A)**, MBR **(B)**, MBD **(C)** n° of spike out of burst **(D)**. Data were normalized with respect to the value at *D0* and subsequently averaged; Comparison between control (*n*° samples = 4) and SKBR-3 (*n*° samples = 4) conditioned medium cell culture: (*) *p* ≤ 0.0001; multiple comparison during the time in culture within control groups (■) *p* ≤ 0.0001 and in SKBR-3 groups (●) *p* ≤ 0.0001.

Figure 3 shows the electrophysiological parameters comparison between control samples and *SKBR-3* conditioned neuronal networks. Both neuronal networks showed a similar trend of MFR during the period in culture; we can observe differences at various time points (Figure 3A). Specifically, starting from *D9*, *SKBR-3* conditioned networks showed higher value of MFR than the *control* ones: 1.34 ± 0.036 (spikes/s) vs. 0.93 ± 0.038 (spikes/s)*,* respectively (*p* < 0.0001). This different behavior was maintained until *D16* (*Ctr* = 0.75 ± 0.031 spikes/s) vs. *SKBR-3* = 1.16 ± 0.058 spikes/s) (*p* < 0.0001). Regarding the bursting behavior, both networks showed predominantly synchronous activity, but the MBR values of *SKBR-3* conditioned networks increased during time (Figure 3B), showing a higher value starting from *D9* (1.50 ± 0.02 bursts/min) from the *control* one (1.06 ± 0.02 bursts/min) (*p* < 0.0001). Furthermore, *SKBR-3* conditioned networks exhibited a MBD (0.75 ± 0.015 s) lower than the *control* ones (0.82 ± 0.017 s) until *D5* (*p* < 0.0001) (Figure 3C); after that, an increase in burst duration was observed starting from *D9* until *D16*, *control* = 0.63 ± 0.019 s) and *SKBR-3* = 0.76 ± 0.002 s), respectively (*p* < 0.0001). Consequently, during the first day after condition, SKBR-3 showed significant higher values of spikes out of burst respect to *control* ones (Figure 3D); then, starting from D9 a change in trend was observed, the control showed higher values than *SKBR-3* ones (*control* = 0.94 ± 0.077 and *SKBR-3* = 0.63 ± 0.037 (*p* < 0.0001)).

**FIGURE 4:**
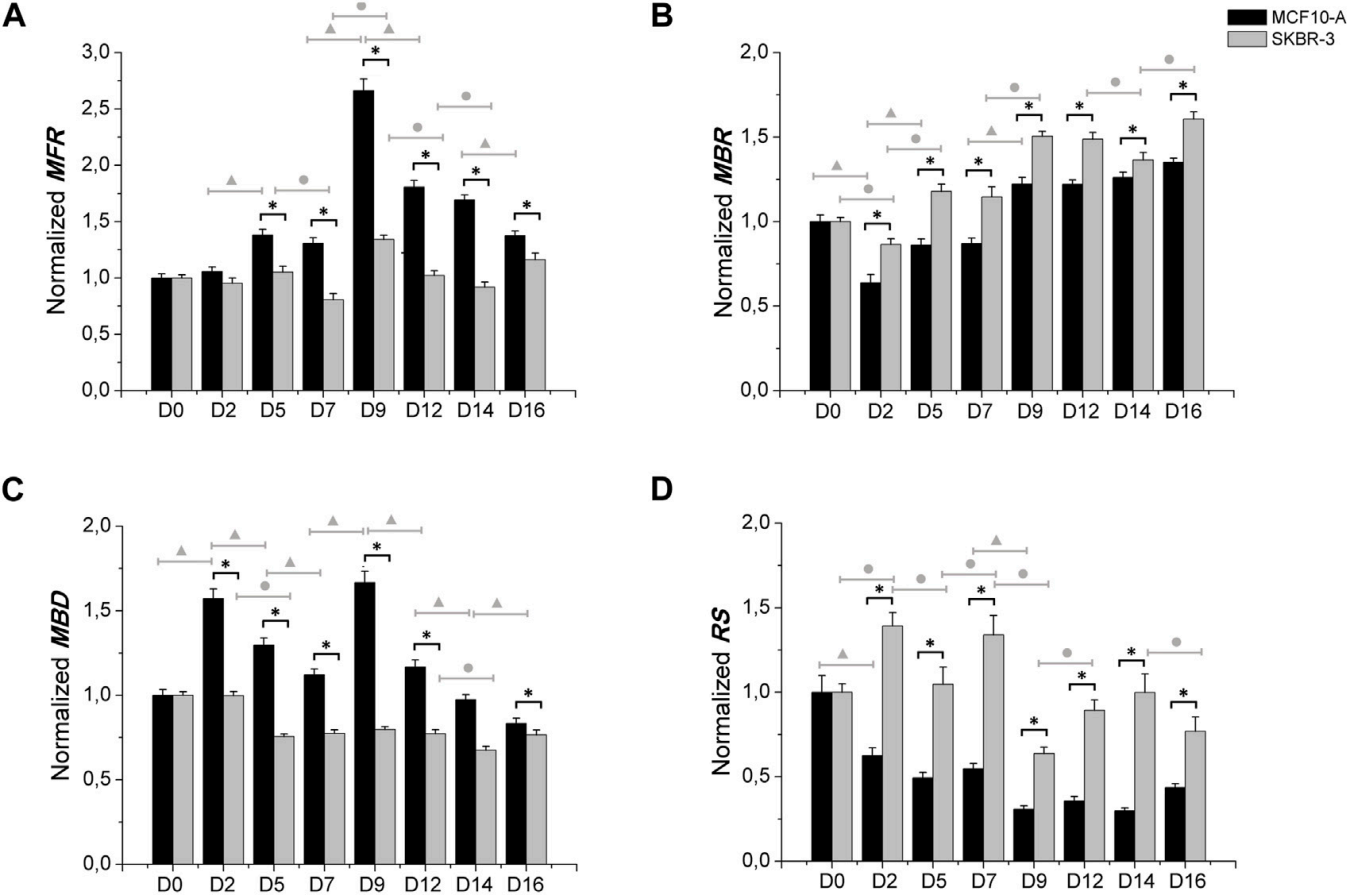
Electrophysiological characterization. MFR **(A)**, MBR **(B)**, MBD **(C)** n° of spike out of burst **(D)**. Data were normalized with respect to the value at *D0* and subsequently averaged; Comparison between *MCF10-A* (n° samples = 4) and *SKBR-3* (n° samples = 4) conditioned medium cell culture: (*) *p* ≤ 0.0001; multiple comparison during the time in culture within *MCF10-A* groups (▲) *p* ≤ 0.0001 and in SKBR-3 groups (●) *p* ≤ 0.0001.

Moreover, the comparison between *MCF10-A* and *SKBR-3* conditioned neuronal networks was carried out, as shown in Figure 4. *MCF10-A* conditioned networks showed higher values of firing frequency compared to S*KBR-3* ones*,* starting from *D5* ((*MCF10-A* = 1.38 ± 0.050 spikes/s) vs. *SKBR-3* = 1.05 ± 0.052 spikes/s), *p* < 0.0001) with the highest values at *D9* (*MCF10-A* = 2.66 ± 0.105 (spikes/s) vs. *SKBR-3* = 1.34 ± 0.036 (spikes/s), *p* < 0.0001) as reported in Figure 4A. In terms of bursting behavior, the MBR of *MCF10-A* conditioned networks showed lower values over time in culture compared to S*KBR-3* conditioned networks (*p* < 0.0001) (Figure 4B). Consequently, *MCF10-A* conditioned networks exhibited higher values of MBD, particularly at *D9* (1.66 ± 0.067 s) which was significantly higher than S*KBR-3* conditioned networks (0.79 ± 0.016 s) (*p* < 0.0001) (Figure 4C). Finally, *SKBR-3* conditioned networks showed significantly higher values of random spikes compared to *MCF10-A* ones (Figure 4D). Moreover, also a quantitative comparison of electrophysiological behavior between the *MCF10-A* conditioned networks and the *control* networks was carried out (Supplementary Figure S3). Furthermore, based on the statistical analysis within individual groups over time, it has been observed that the *control* group did not exhibit statistically significant differences during the recording days. This suggests the formation of mature and stable neuronal cultures over time (Figure 3). On the other hand, a persistent statistical difference across various days was observed in different parameters (MFR, MBR, and RS) throughout the recording period for both treatments with conditioned *SKBR-3* and *MCF10-A* media (Figure 4). The results were more evident in *SKBR-3* than in *MCF10-A* conditioned cultures; *MCF10-A* conditioned networks, after an initial perturbation, showed a more stable bursting behavior and lower random spiking activity than *SKBR-3.* Whereas *SKBR-3* conditioned networks showed an increase in MBR and RS values over time, higher than both control and *MCF10-A* groups. Finally, significant statistical differences were observed in both treatments regarding MFR and MBD values over the time. These results suggest that both conditioned media induced a functional perturbation, even if expressed only in some parameters, probably due to the high sensitivity of neuronal cells.

### 3.2 Morphological characterization

**FIGURE 5:**
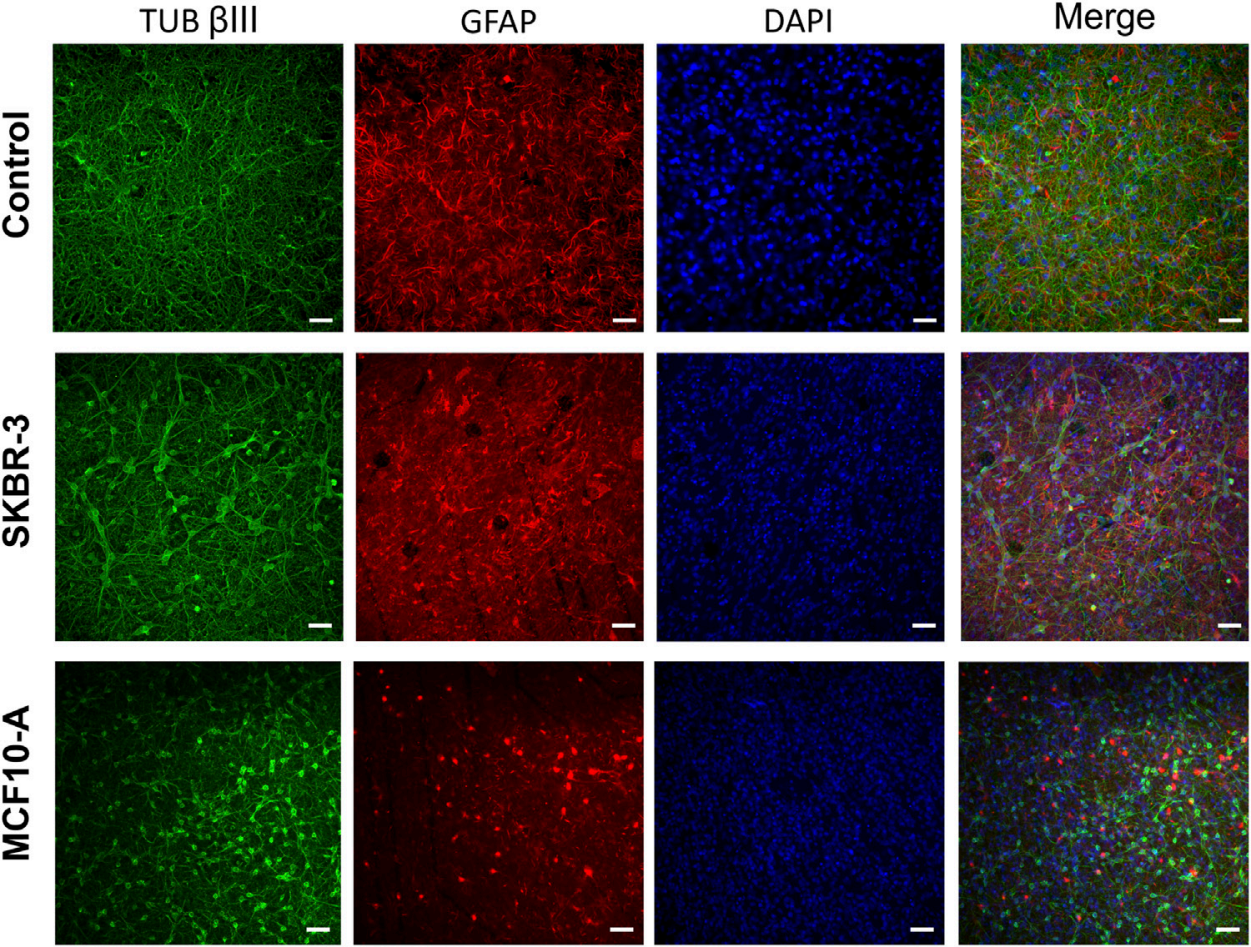
Optical images of 2D neuronal cultures stained for TUB βIII (green), GFAP (red) and DAPI (blue) at DIV57. Scale bar: 20 μm.

To evaluate the effect of *conditioned medium* produced by the cell lines *SKBR-3* and *MCF10-A* on nervous cells, neurons (labeled with anti-Tub ßIII antibodies) and astrocytes (labeled with anti-GFAP antibodies), a qualitative morphological analysis was carried out. All samples were fixed at DIV 57 (*D16*). Immunofluorescence labeling showed striking differences in neuronal morphologies in the three conditions; specifically, in CTR neuronal cells displayed a fine structured and extended network, while both *SKBR-3* and *MCF10-A* conditioned neuronal cells exhibited a coarsely structured and less extended network (Figure 5). Additionally, the conditioned neuronal networks, especially those conditioned with *MCF10-A* conditioned medium, showed a change in cell body morphology resembling a hypertrophic shape (Supplementary Figure S4). Ultrastructural examination of both *SKBR-3* and *MCF10-A* conditioned neuronal cells showed the presence of a dense network with several connections and conventional synapses. No remarkable morphological differences were observed between these two conditions (Supplementary Figure S5).

**FIGURE 6:**
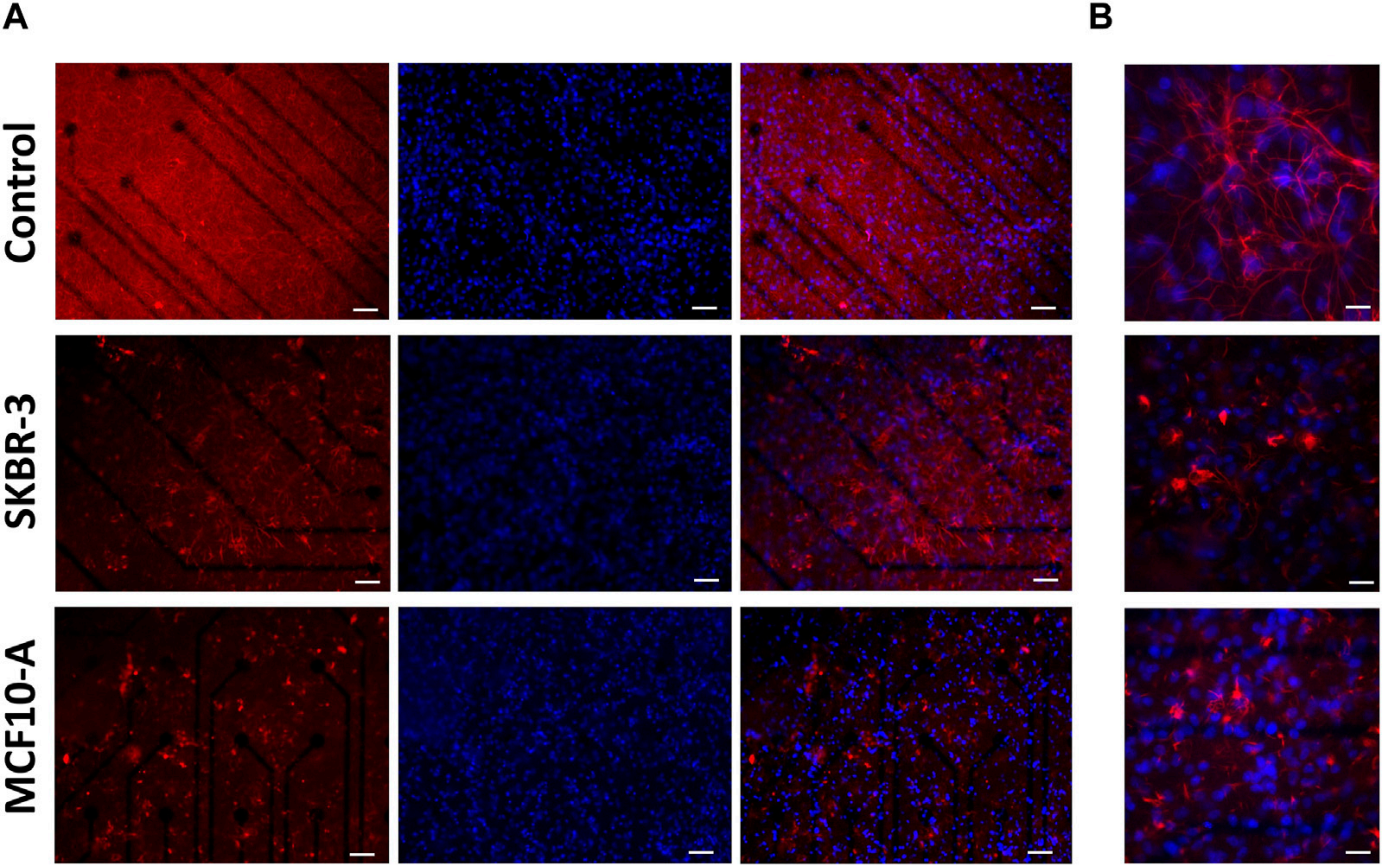
Optical images of 2D neuronal cultures stained for GFAP (red) and Dapi (blue) at DIV57. Scale bar: **(A)** 50 μm and **(B)** 20 μm.

Regarding the glial fraction, in the *control* networks, astrocytes displayed a conventional morphology (Tedesco et al., 2018), that is a highly ramified with extensive arborizations, while the conditioned cultures exhibited a modified morphology characterized by increased heterogeneity and loss of the normal structure (Figure 6A). At higher magnification, it was possible to appreciate and highlight the hypertrophic characteristics of astrocytes (Figure 6B). Indeed, the size of astrocyte bodies appeared shrinked in comparison to *control*. To quantify this qualitative observation, the fluorescent intensity and the percentage of covered area by GFAP-positive cells were assessed. The results confirmed the change in glial cells morphology (Supplementary Figure S6). Previous studies have reported similar results, demonstrating the modified morphological behavior of glial cells in response to the presence of metastatic breast cancer cells (Fitzgerald et al., 2008; Neman et al., 2014).

## 4 Discussion

A deeper understanding of the interactions between breast cancer cells released factors and the brain microenvironment is crucial for the development of more effective treatments for brain metastases. The aim of this study was to explore the influence of conditioned medium, derived from *SKBR-3* cultures (a commonly used ERBB2+ human breast cancer cell line) and *MCF10-A* (a non-tumorigenic human mammary cell line), on both the electrophysiological activity and morphology of neural networks. Our findings revealed that the conditioned medium from ERBB2+ SKBR3 cells and non-tumorigenic *MCF10-A* had distinct effects on electrical activity and the morphology of neurons and astrocytes.

### 4.1 Electrophysiological characterization

To evaluate the effects of *SKBR-3* and *MCF10-A* cell conditioned medium (vesicles and soluble factors) on nervous cells, electrophysiological analyses were performed. The long-term electrophysiological impacts of exposing a co-culture of hiPSC-derived neurons and rat astrocytes to the *conditioned medium* were investigated using the MEA system. During the experiment, samples were subjected for 2 weeks to the medium conditioned by *SKBR-3* and *MCF10-A* cells. A control group was also included, consisting of iNeurons-astrocytes co-cultured in *iNeurons medium*, as depicted in Figure 1. Quantitative parameters representing the behavior and functionality of the neuronal networks were derived from recorded spontaneous electrophysiological activity. These parameters were compared to determine whether conditioning induced any modifications in the physiological behavior of the network. Firstly, functional alterations were observed in all three conditions at *D2*, which is a common occurrence following medium changes. Therefore, the focus of characterization was primarily directed towards observing the effects 1 week after conditioning, allowing the networks to stabilize within the altered environment. The activity exhibited by *SKBR-3* conditioned networks showed fluctuations influenced by the initial conditions, indicating a general increase in activity at *D9*. The statistical difference between *SKBR-3* conditioned network and the control ones is highly significant. In the case of *MCF10-A, a* considerable increase in spiking activity, resulting in a firing frequency, was observed. *SKBR-3* conditioned networks demonstrated an increase in bursting activity, consistent with qualitative observations of raster plots. Specifically, from *D9* onwards, *SKBR-3* conditioned networks exhibited a statistically significant increase in activity compared to the control ones. The *control* networks maintained a consistent trend with no significant changes, while *MCF10*-A conditioned ones, following a more critical initial adjustment, demonstrated patterns similar to those observed in the *SKBR-3* conditioned samples. Burst activity is one of the main features of the development of network functionality (Wagenaar et al., 2006; Odawara et al., 2016) and efficient information conduction within the brain under normal physiological conditions, (Khazipov and Luhmann, 2006; Wagenaar et al., 2006; Egorov and Draguhn, 2013; Odawara et al., 2016); iNeuronal networks, after DIV41, should exhibit consistent and synchronous burst production frequencies. Bursting activity *in vitro* plays a crucial role in establishing functional connections between neurons and facilitating coordinated activity. However, uncontrolled bursting behavior or heavy strong bursting are considered a characteristic feature of the epileptic brain: in fact, they are also considered as seizure-like events in MEA recordings (Odawara et al., 2016; Chong et al., 2018; Liu et al., 2018). Specifically, breast cancer is considered a prominent factor in the development of epilepsy originating from brain tumors (Cosgrove et al., 2022). Epilepsy is characterized by abnormal neuronal activity, wherein the occurrence, spread, and cessation of epileptic seizures depend on interconnected networks of neurons that engage in synaptic and non-synaptic interactions (Wu et al., 2015). In epilepsy, the synchronized bursts of neuronal activity become excessive, unregulated, and disruptive, leading to abnormal electrical discharges and potential seizure events (Odawara et al., 2016). The increase in burst frequency, as well as the presence of disorganized and uncontrolled functional events observed in neuronal networks conditioned by SKBR-3 cells, aligns with the results reported in the existing literature regarding epilepsy events. These findings provide further support and can be explained by the previous studies conducted in this field, suggesting that epileptic events may be linked or induced by the tumor’s conditioned medium. Additionally, the analysis of mean burst duration (MBD) demonstrated a consistent decreasing trend in all samples. In the control networks and *SKBR-3* conditioned ones, the values of MBD showed a tendency to stabilize over the observation period. This suggests that the electrical activity or the specific parameter being measured reached a relatively steady state in these groups. The decrease in burst duration observed confirmed the previously detected increase in burst frequency in networks conditioned by *SKBR-3*. However, in the *MCF10-A* conditioned networks higher values were observed compared to the other two groups of networks. The control ones maintained a consistent electrophysiological trend, with minor random variations in spike activity compared to *D0*. On the other hand, *SKBR-3* conditioned networks exhibited significant oscillations around the initial value. The observed changes were described as oscillating periods, with distinct characteristics. Specifically, the neuronal activity exhibited highly synchronous periods, demonstrating a more coherent and structured pattern. These synchronous periods alternated with periods of disorganization, where the activity became more irregular. Supplementary Figure S2 (Supplementary Material) provides further evidence and visual representation of these alternating patterns. Meanwhile, *MCF10-A* conditioned networks demonstrated a reorganization and maintenance of network activity.

Finally, electrophysiological data indicated that the neuronal networks exhibited a substantial and consistent reaction to the *conditioned medium* of both the *SKBR-3* cell line (a breast cancer cell line) and the *MCF10-*A mammary non-tumorigenic cell line. The results suggest that the secreted factors from both cell lines have a significant impact on the functional behavior of the nervous model, potentially indicating their influence on cellular communication or signaling pathways. However, differences in electrophysiological behavior between *SKBR-3* conditioned networks and *MCF10-A* suggest that neuronal dysfunction could potentially arise from factors not only attributed to the mass effect of the metastases, but also from substances secreted by cells in the tumor microenvironment. The observed changes in excitability and electrophysiological signals resembled interictal-like epileptic activity (Holmes and Lenck-Santini, 2006; Simon et al., 2020). The correlation between tumor growth and the development of epilepsy has been widely studied in the medical field. As it has already been observed in literature, changes in electrophysiological parameters have been linked to cognitive impairment in patients with epilepsy (Pelkonen et al., 2020). This similarity suggests that our model may capture early stages of nervous tissue disorganization, which could potentially lead to the development of tumor-associated epilepsy. Altered expression and/or activity of neurotransmitter receptors and ion channels can be responsible for inducing this specific activity, as demonstrated in previous research (Lévesque et al., 2018; Simon et al., 2020). Overall, these alterations contribute to the enhancement of glutamatergic transmission while reducing GABAergic activity. Furthermore, neuronal atrophy and/or disrupted connectivity have been observed also in chronic phases of rodent epilepsy models, (Lévesque et al., 2018). However, it is important to note that further investigation and analysis are necessary to fully understand the underlying mechanisms and implications of this alteration in electrophysiological activity.

### 4.2 Morphological characterization

The immunocytochemistry characterization allowed us to gain information on the morphology of the 2D neuronal networks after different conditioning. In *SKBR-3* and *MCF10-A* conditioned networks, neurons showed a hypertrophic morphology and the formation of less dense networks, suggesting unhealthy conditions. Remarkable changes in astrocyte morphology were observed in response to the presence of breast cancer-conditioned medium, showing a significant reduction in ramification compared to the control networks. These results confirmed that an alteration of normal conditions occurred, considering that under normal physiological conditions, glial cells usually exhibit a highly ramified morphology (Fitzgerald et al., 2008; Neman et al., 2014). Moreover, also astrocytes showed a hypertrophic morphology after conditioning. Interestingly, a similar result was observed in patients undergoing neurosurgical resection of breast-to-brain metastasis (Neman et al., 2014). Neman et al. demonstrated that metastatic breast cancer cells were juxtaposed to reactive astrocytes expressing glial fibrillary acidic protein (GFAP) both in the peritumoral brain and tumor regions, which showed a hypertrophic morphology comparable to astrocytes in glial scars forming in response to traumatic brain injury (Herrmann et al., 2008). Studies have demonstrated that astrocyte activation in the presence of pathological conditions like trauma, ischemia, and neurodegenerative diseases can be a protective mechanism for neurons, preventing injury-induced apoptosis (Neman et al., 2013). Furthermore, astrocytes play vital roles in various aspects of neural function, promoting nutrients transportation to neurons and oligodendrocytes. Additionally, astrocytes are involved in neural signal transduction, supporting the transmission of signals between neurons and the maintenance of the ionic balance of the extracellular matrix. In conclusion, alterations in astrocytes can be directly linked to neuronal electrophysiological activity; therefore, morphological results of this study can support, explain, and justify the electrophysiological ones.

## 5 Conclusion

This study aimed to assess the influence of medium conditioned by breast cancer cells on neurons and astrocytes. We characterized the impact of factors released from a cell model of ERBB2+ breast cancer on the electrophysiological activity and on the morphology of mature neuronal networks. Our results clearly demonstrated that the *conditioned medium* modifies the activity of the network in terms of both spiking and networking. Morphological alterations also supported the observed functional evidence. Specifically, the apparently morphological alteration of astrocytes, considering their importance for the functional activity of the network, are thought to play a fundamental role in the modification of the network itself. In addition, similar structural changes have been previously observed *in vivo* in activated astrocytes and glial scars. In this work, we reported the first evidence, to the best of our knowledge, of *in vitro* electrophysiological activity alteration of neuronal networks by breast cancer cells. Our results represent a first step toward understanding central nervous system functional alterations promoted by factors released by breast cancer metastases. The next step would be to break down the components of the factors released by breast cancer cells to assess which is responsible for the observed neuronal and glial alterations.

## Data Availability

The raw data supporting the conclusion of this article will be made available by the authors, without undue reservation.
