# Supplementary material for: Electrophysiological and morphological modulation of neuronal-glial network by breast cancer and nontumorigenic mammary cell conditioned medium
Source: Front Bioeng Biotechnol. 2024 Apr 4;12:1368851. doi: 10.3389/fbioe.2024.1368851 (PMC11024227; doi:10.3389/fbioe.2024.1368851)
Supplement: Supplementary file 1 [file DataSheet1.docx]

Electrophysiological and morphological modulation of neuronal-glial network by breast cancer and nontumorigenic mammary cell conditioned medium

**Donatella Di Lisa^1,3^, Katia Cortese^2^, Michela Chiappalone^1,3,4^, Pietro Arnaldi ^2^, Sergio Martinoia^1,4^, Patrizio Castagnola^3^, Laura Pastorino^1,3,4^**

^1^ DIBRIS, Department of Informatics, Bioengineering, Robotics and Systems Engineering, University of Genoa, Via Opera Pia 13, 16145 Genoa, Italy.

^2^ DIMES, Department of Experimental Medicine, Cellular Electron Microscopy Lab, University of Genoa, Via Antonio de Toni 14, 16132, Genova, Italy.

^3^ IRCCS Ospedale Policlinico San Martino, Largo Rosanna Benzi 10, 16132, Genova, Italy

^4^ RAISE Ecosystem, Genova, Italy'

Supplementary Material

**Supplementary Figure S1**


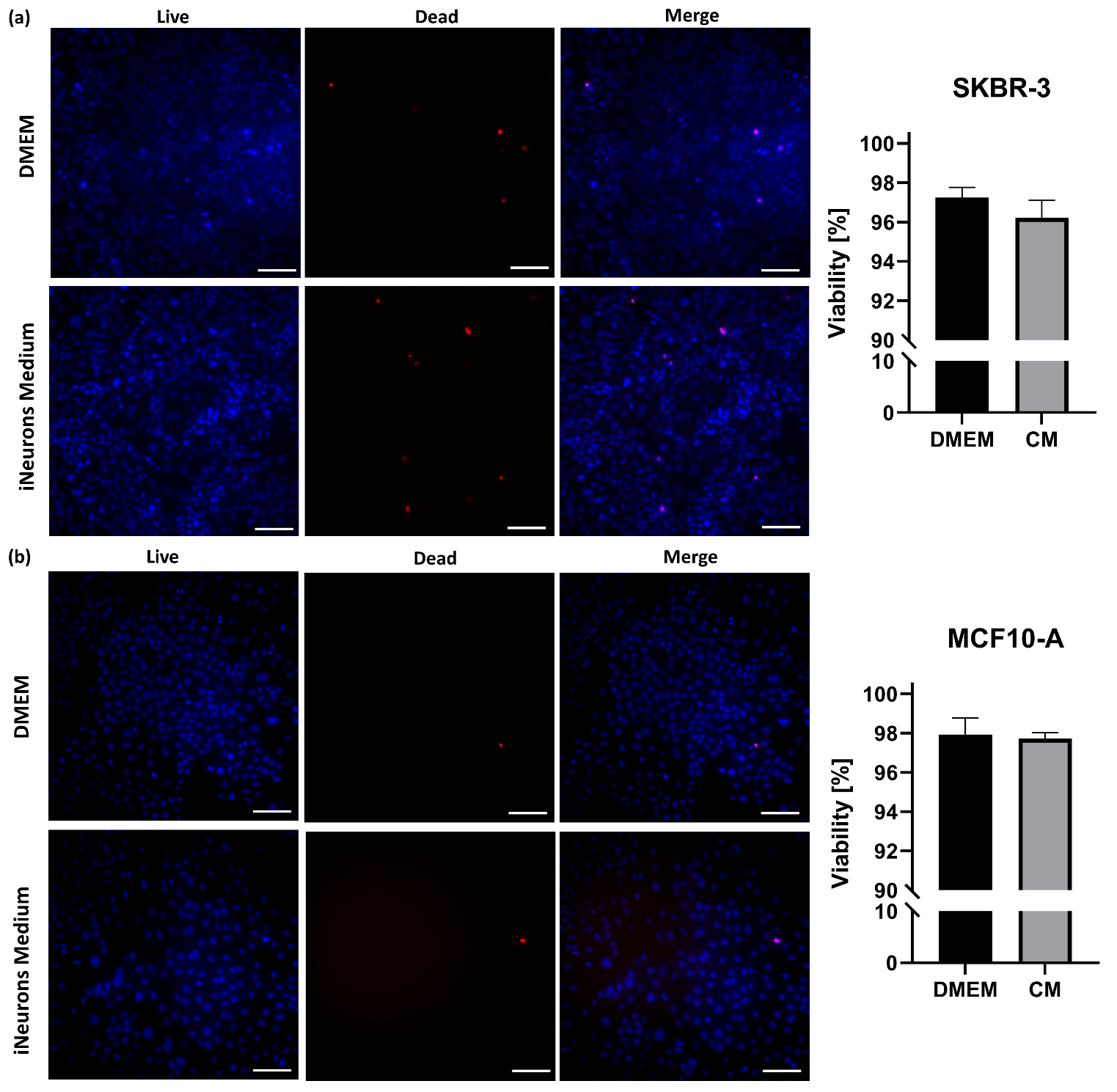


**Supplementary figure S1. Viability assay of human breast cell lines.** Live/dead assay, discriminating live cells (blue labeled) and dead cells (red labeled) in SKBR-3 (a) and MCF-10A (b) cultures after 48 h exposure to standard culture medium (DMEM) and conditioning medium (iNeurons Medium), respectively. Scale bar = 100 µm. Right panel: Bar plot showing the percentage of live cells to total cell populations (N=3 samples per condition).

**Supplementary Figure S2**


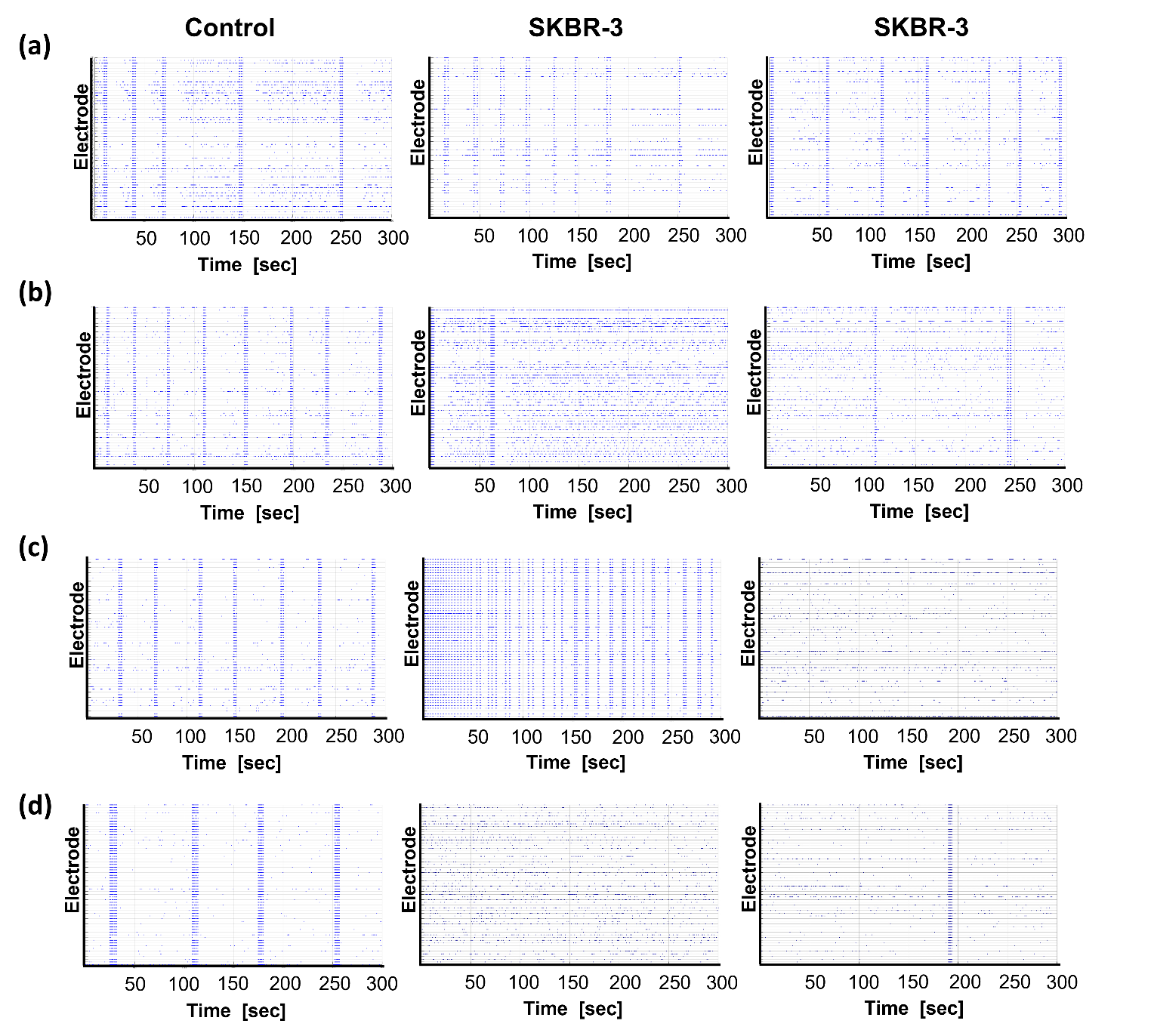


**Supplementary figure S2. Spontaneous activity characterization.** Raster plot showing 300 s of spontaneous activity of 2D control network and SKBR3 conditioned neuronal networks at different time points during development (**a**) D0, (b) D2, (**c**) D9, (**d**) D16.

**Supplementary Figure S3**


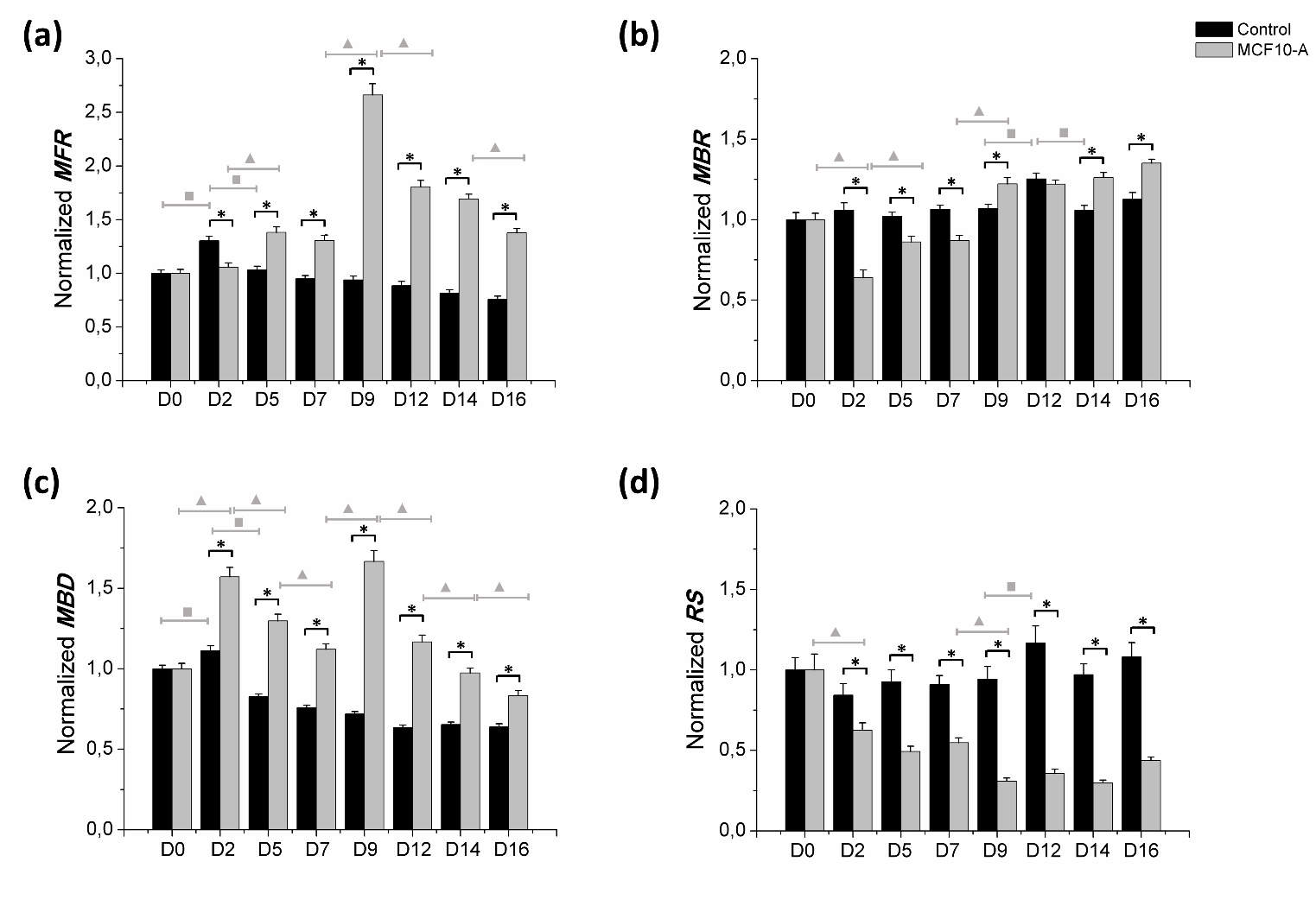


**Supplementary figure S3. Electrophysiological characterization.** MFR **(a),** MBR **(b),** MBD **(c)** n° of spike out of burst **(d).** Data were normalized with respect to the value at D0 and subsequently averaged. Comparison between control and *MCF10-A* conditioned medium cell culture, (*) P ≤ 0.001 and multiple comparison during the time in culture in *MCF10-A* groups (▲) P ≤ 0.001 and in SKBR-3 groups (●) P ≤ 0.001.

**Supplementary Figure S4**


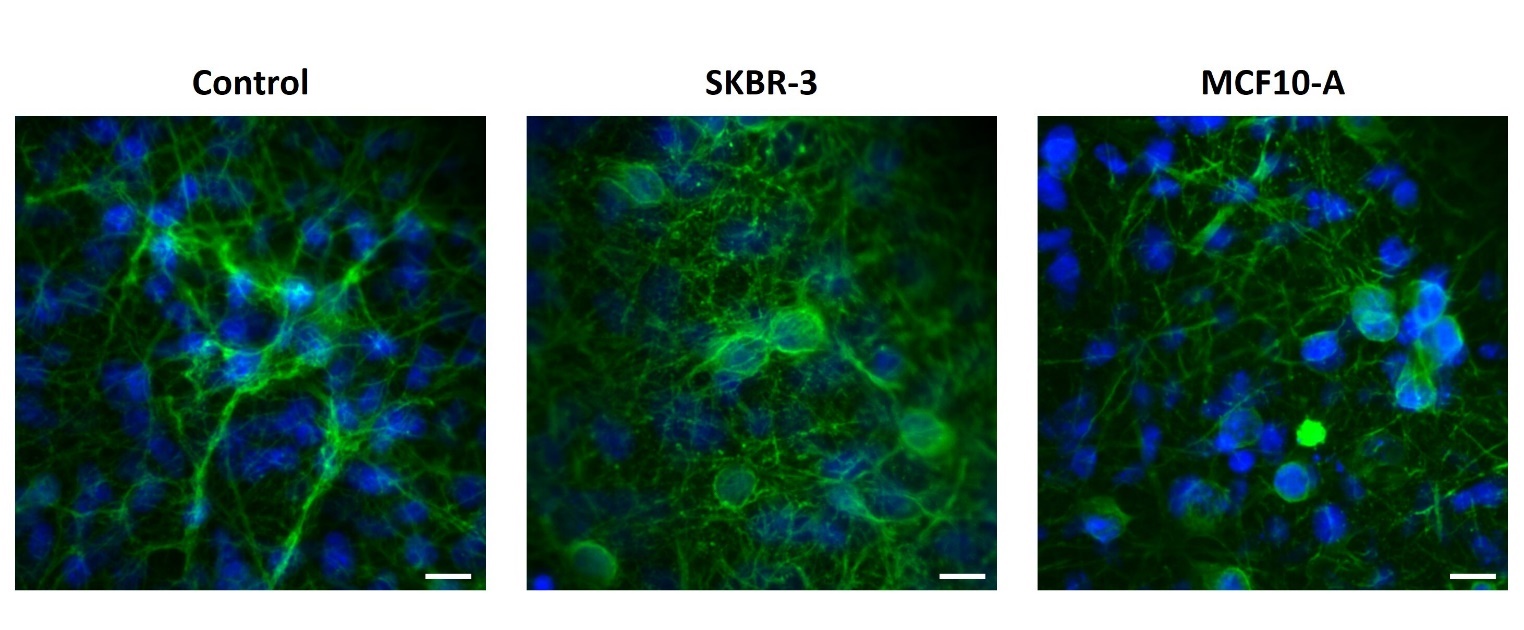


**Supplementary figure S4. Optical fluorescent images.** 2D neuronal cells stained for TUB βIII (green) and DAPI (blue) at DIV57. Scale bar: 20 μm.

**Supplementary Figure S5**


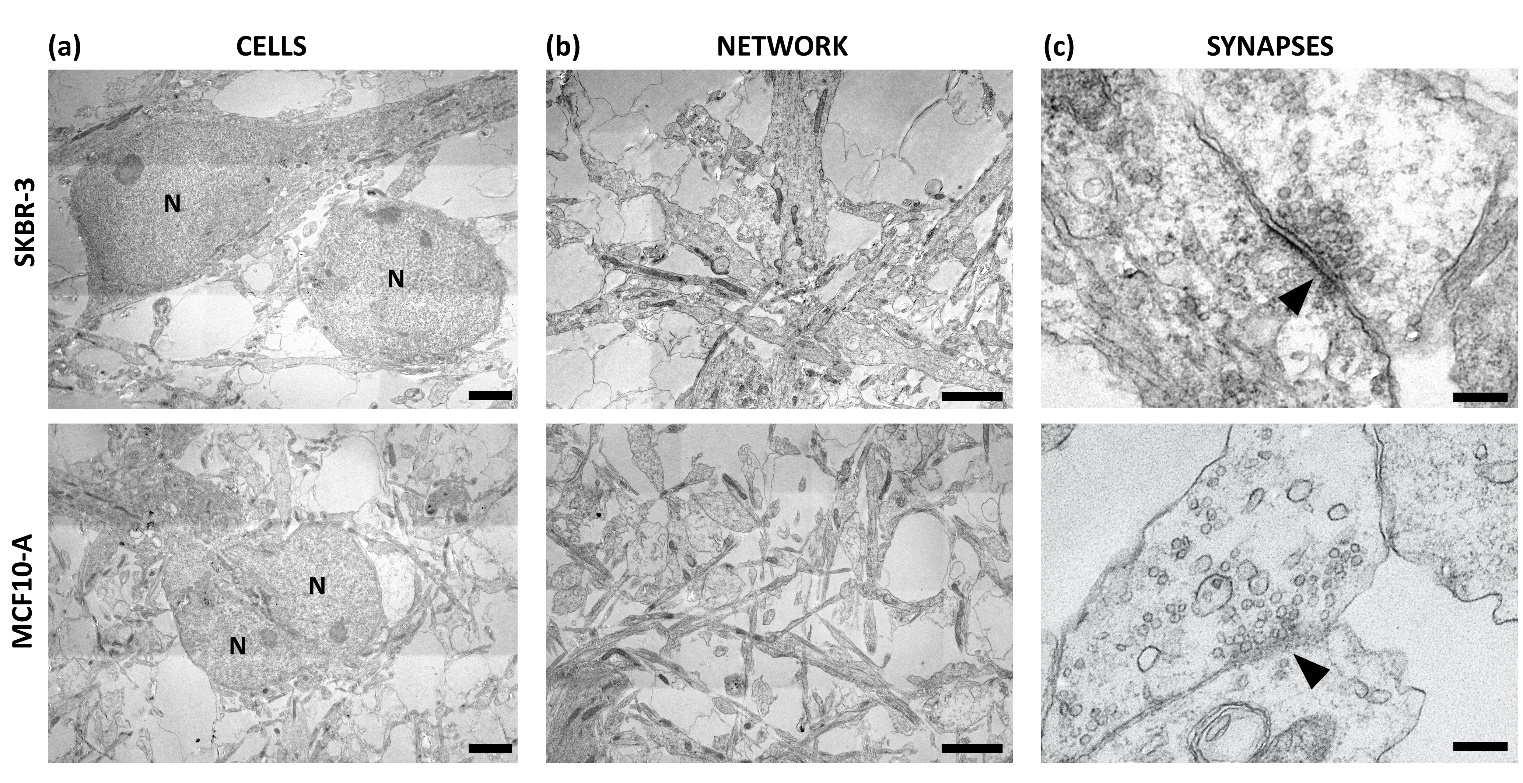


**Supplementary figure S5. Ultrastructural characterization.** Representative TEM micrographs of SKBR3 and MCF10A conditioned neuronal networks on the final day of conditioning (D16). (**a**) Cell bodies detail (N: Nucleus, scale bar: 2 µm). **(b)** Neural-glial dense network detail (scale bar: 2 µm). (**c**) High-magnification detail of synapses indicated by black arrowheads (scale bar: 200 nm).

**Supplementary Figure S6**


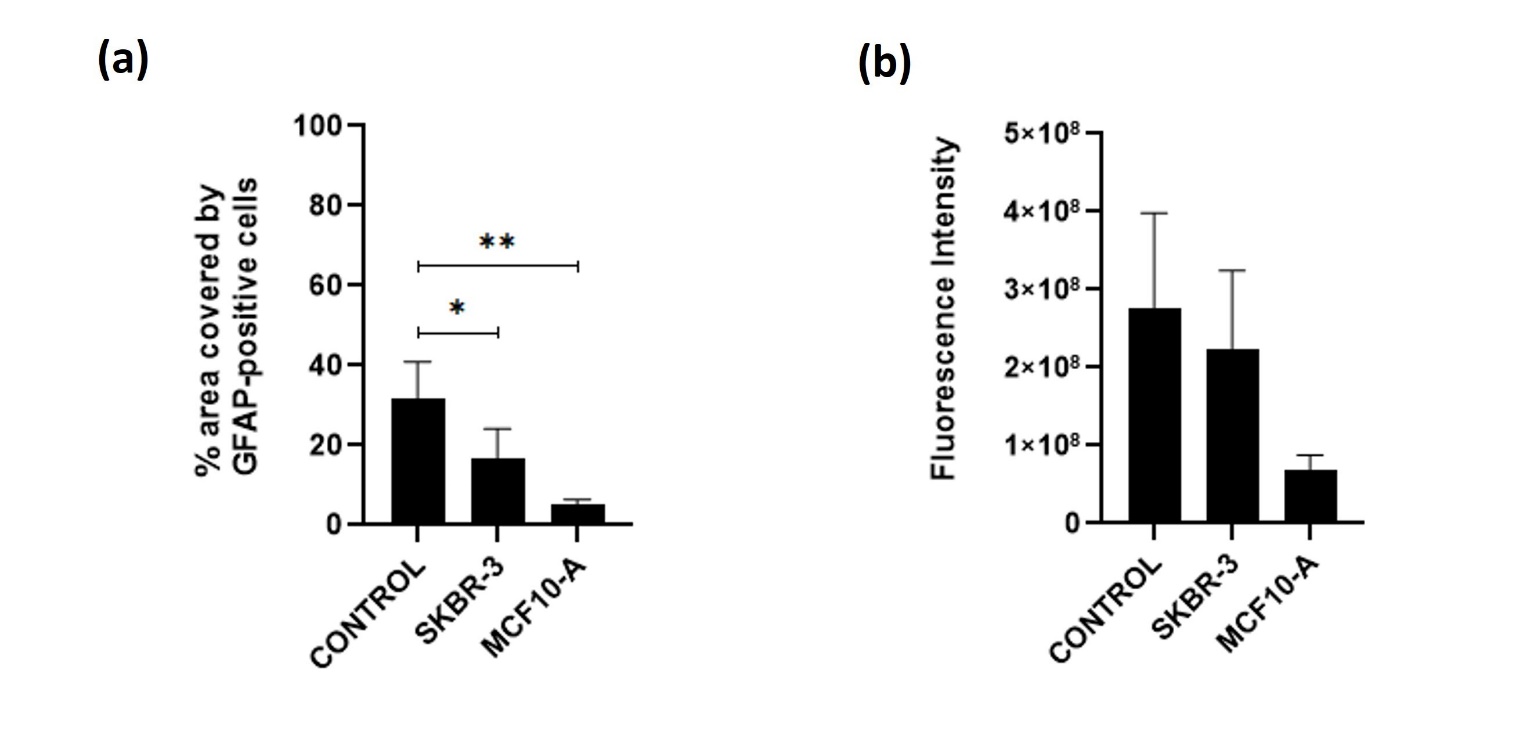


**Supplementary figure S6. GFAP-positive cell morphology characterization.** (**a**) Percentage of covered area by cells expressing GFAP, (**b**) fluorescence intensity.
